# A New Theory as to the Etiology of Pellagra

**Published:** 1913-01

**Authors:** Roy Blosser

**Affiliations:** Atlanta, Ga.; 403 Atlanta National Bank Building


### A NEW THEORY AS TO THE ETIOLOGY OF PELLAGRA

By Roy Blosser, M. D., Atlanta, Ga.

In the December number of the Journal-Record I made a preliminary report on the treatment of pellagra by the internal administration of gelsemium. I have now treated a number of other cases in this way with the most gratifying results and shall report them in the near future.

In studying the dietetic habits of these patients I observed that one of the most constant faults was the habit of eating large quantities of cane syrup and candy. In this connection I have questioned twenty-two pellagra cases regarding their use of these sweets and there has been a striking unanimity in the replies which I have received. Every one of these cases had in the past been very free users of syrups and candy—possessors of what is commonly known as a "sweet tooth." Over half of them stated that they had found that they had to discontinue the use of syrup, most of them because it caused "burning" in the stomach, and others noticed that it produced nervous excitement and palpitation of the heart.

The above facts suggested to my mind a new theory as to the etiology of this disease, namely, the excessive use of cane-sugar by-products.

As is well known, pellagra is much more prevalent in the South than in the North and a corresponding fact is that sugar cane syrup has long been a regular article of diet in the South.

It was formerly made by almost every farmer by the old open kettle method, which has more recently been supplanted by the centrifugal and vacuum pan method.

There are several reasons why the people of the South depend so largely upon cane syrup and other sweets as a necessary part of their diet. As is generally known, the Southern people are exceedingly fond of hot biscuit, hot griddle cakes and waffles. In many homes these are eaten three times a day, and always with large quantities of syrup, especially in the spring and early summer, when some other foods are less abundant.

A striking fact is brought out by a comparison of two maps issued by the Department of Agriculture and the U. S. Public Health Service, namely, that the zone of greatest pellagra prevalence in the United States is just south of the zone of beet sugar production. The southern limit of this latter zone corresponds to a line through the center of Pennsylvania, Ohio, Indiana, Illinois and Iowa; it then dips south to the western part of Texas and continues west to the coast. This line is closely paralleled by the northern limit of the zone of greatest pellagra prevalence. Furthermore, those states in which pellagra does not occur are north of the beet sugar section and therefore fartherest removed from the cane sugar sction. Of course these cane syrups and mixtures are now sold in every part of the country, but the South is by far the largest users of them—a habit established when nearly every farmer raised a crop of sugar-cane for making syrup.

Another pertinent fact is that the present epidemic of pellagra has followed the introduction of the modern method of sugar manufacture with the vacuum pan and centrifugal machine. The latter superceded the old open-kettle process. The explanation of the increased prevalence of the disease following this change in the method of manufacture may be found in the fact that while by the new method, a much larger per cent. of crystallizable sugar is obtained, the residue which is made into commercial cane syrup, contains a much larger per cent. of the non-crystallizable solids inherent in sugar cane. Sugar cane juice as it comes from the cane stalks contains an average of 16 ½% of solids. The manufacturers are now able to secure an average of about 14½% of crystallized sugar from the cane juice, which deducted from the average solids leaves 2% of non-crystalizable solids for making syrup. Considering that the average syrup contains 75% of solids, we get an idea of the amount of evaporation and concentration which has taken place. The purest part has been removed—granulated sugar being generally over 99% pure—and we have a residue of very concentrated extract of sugar cane.

This is mixed with glucose to prevent its having too strong a flavor, and sold as sugar cane syrup, or it is bleached by chemicals which gives the syrup a pale straw color. It is a very different syrup however from that manufactured by the old open-kettle method on account of having a much larger percentage of non-crystallizable solids. The old fashioned brown sugar also contains a considerable amount of these non-crystallizable solids, but there is not much of it sold now as it is more profitable, by further refining, to produce white sugar.

Of interest in this connection is the fact brought to my attention by Dr. L. P. Pharr that the symptoms of beriberi show a number of points of resemblance to those of pellagra; also that it is now practically proven that beriberi is due to an excessive carbohydrate diet in the form of rice. There has been a remarkable diminution in the occurrence of this disease n the Japanese navy since the introduction by Takagi of a diet allowing a larger proportion of nitrogenous food. The following symptoms occur in this disease (Osler): Pain, muscular weakness, lowering ot sensibility in the legs, and variously located paresthesiae; also, palpitation of the heart and uneasy sensations in the abdomen. After lasting for a variable time these symptoms may all disappear, *but with the return of warm weather there may be a recurrence* Thus there is seen to be a striking analogy between the symptoms of these two diseases, their season of greatest serverity, the former that both occur in warm countries, and the etiology of the farmer as compared with this theoretic etiology of pellagra.

As to the use of sugar-cane products in Italy I am unable to obtain any reliable information except that sugar-cane is grown in some parts of that country. In Spain there has been a marked decrease in the number of cases of pellagra in recent years and during the same time the amount of the sugar-cane products consumed has greatly diminished for the following reason: prior to 1891 the enormous sugar-cane production of Cuba was practically all exported to Spain. In this year, on account of new trade relations, the United States began getting an increasing amount of these exports; since gaining possession of the island practically all of the sugar-cane products are disposed of in this country. The same thing applies to Porto Rico. The latter island produces about the same amount of sugar-cane as the United States; Cuba produces five or six times as much, or about one-fourth of the sugar-cane crop of the world. Thus we see that coincident with the decrease of pellagra in Spain and the increase of the disease in this country the enormous sugar-cane output has been largely shifted from Spain to this country.

While it will naturally require much more conclusive experimentation and investigation than is offered in this paper to prove that sugar-cane products can cause pellagra, it seems reasonable to the author to believe that this is a possible explanation of the etiology of the disease, and further, that the recent terrible increase of pellagra in the South may be due to the change in the process of refining sugar and the use of the concentrated by-products for making syrups and the large consumption of these syrups and other sweets made from them. According to this theory the frequency of pellagra among poor people may be accounted for by the fact that, on account of the cheapness of these syrups, they are very largely used by this class. The explanation of its prevalence among the rural population may be found in the fact that these people are not in easy reach of good markets and therefore, especially in the winter and spring when their meat supply—mostly pork—is apt to become exhausted and fruits and vegetables are out of season, they depend largely on these syrups to supply the deficiency.

403 Atlanta National Bank Building.

After writing the above paper it was brought to my attention that Dr. Deeks of the Canal Zone had advanced a similar theory in the Medical Record of March 23rd, 1912. Dr. Deek's idea is that pellagra is caused by a diet containing insufficient proteids, green vegetables and fruits, too much cane sugar and starchy foods, and the lessened digestive and metabolic activities for this latter class of foods incident to living in a warm climate.

